# Addressing the Adult Soft Tissue Sarcoma Microenvironment with Intratumoral Immunotherapy

**DOI:** 10.1155/2018/9305294

**Published:** 2018-08-12

**Authors:** Shailaja Raj, Lance D. Miller, Pierre L. Triozzi

**Affiliations:** Wake Forest School of Medicine, Winston-Salem, NC, USA

## Abstract

Sarcoma is comprised of a heterogeneous group of tumors originating from the mesenchyme. Sarcoma is also the first tumor that responded to immunotherapeutic agents often termed as “Coley's toxins.” However, immunotherapy is yet to establish its presence in sarcomas. Complex interactions between tumor and immune cells in the tumor microenvironment play a crucial role in response to immunotherapy. There is a dynamic equilibrium created by the immune cells infiltrating the tumor, and this forms the basis of tumor evasion. Manipulating the intratumoral microenvironment will help overcome tumor evasion.

## 1. Introduction

In this review, we first explore the specific oncogenic alteration characteristics of different subtypes of sarcoma. This is followed by a description of the mechanisms by which tumor infiltrating lymphocytes affect prognosis and the specific immune cell populations that can be targeted and manipulated in different subtypes of sarcoma. We also review the various immune suppressive mechanisms including immune check points, receptors, and tumor-associated macrophages and their relevance in sarcoma. We then focus on intratumoral immunotherapy, mechanisms of immune interactions, limitations, and the types of intratumoral therapies including oncolytic viruses, immune cells, and cytokines. We foresee intratumoral immunotherapies being able to target and influence management of sarcomas in the future.

Soft tissue sarcomas arise from cells of mesenchymal lineage, including muscle, fat, blood vessel, and nerve. Over 50 histological types have been identified. Localized tumors are usually well controlled with surgery. Localized tumors that have high-grade histologies and those are over 5 cm, however, have over a 50% risk of recurrence. Patients with high-risk localized tumors are usually treated with combinations of surgery, radiation, and/or chemotherapy. These approaches have failed to substantially improve overall survival. Chemotherapy has not significantly impacted the outcome of patients with metastatic soft tissue sarcoma. The prognosis of these patients is very poor. Median overall survival is 8 to 12 months [1].

Immunotherapy has been an attractive approach to treat refractory cancers. Sarcoma is considered to be the first cancer for which immunotherapy was effectively applied. Based on observations of tumor regressions in patients with concomitant streptococcal infections, William B. Coley injected streptococcal organisms into tumors, especially sarcomas, in the last decade of the 19th century [2]. Over 50% of the inoperable sarcoma patients who are Coley treated were reported to respond completely. Furthermore, approximately 20% survived over 20 years [2]. With their poorly characterized preparation and unpredictable toxicities, “Coley's toxins” never became clinically useful. Immunotherapeutic approaches have been tested in patients with soft tissue sarcoma. The results have not been as spectacular as some of the other solid tumors. Immune checkpoint blockade with antibodies that target cytotoxic T lymphocyte-associated antigen 4 (CTLA-4) and the programmed cell death protein 1 pathway (PD-1/PD-L1) is leading to durable clinical responses in an increasing number of cancers. However, responses in patients with soft tissue sarcoma have been infrequent [3–6].

Immunotherapy response is dependent on complex interactions between tumor and immune cells within the tumor microenvironment. Several factors determine whether or not immunotherapy response will be promoted or inhibited. These include the inherent antigenicity of the tumor. Mutations in proteins and/or aberrant proteins expressed by tumor cells and the “neoantigens” they generate are the primary targets for T-cell-mediated destruction. Tumor mutation burden has emerged as a quantitative marker that can help predict responses to immune checkpoint inhibition across different cancers. Immunotherapy response is also dependent on the infiltration into tumor of immune effector cells. Specific patterns of tumor infiltrating lymphocytes (TILs) within the tumor microenvironment are associated with improved outcome in patients with many types of cancers, regardless of the type of therapy administered. Most importantly, a variety of processes within the microenvironment can suppress interactions between tumors and immune effector cells and promote the escape of tumors from immune surveillance. Immune checkpoint ligands and receptors have emerged as the major targetable mechanism of tumor immune escape. Several other molecular, soluble, and cellular factors are involved, and whether or not these factors are being addressed will also determine immunotherapy sensitivity.

Understanding the soft tissue sarcoma microenvironment is not only critical relative to improving the efficiency of current immunotherapies but also for the development of more effective approaches. In this review, we focus on the recent advances made in understanding the immune microenvironment in soft tissue sarcoma. We also discuss the rationale for building upon Coley's work and directly modifying the soft tissue sarcoma microenvironment using the intratumoral administration of immunologically active agents.

## 2. Tumor Immune Microenvironment

### 2.1. Tumor Antigenicity

Soft tissue sarcomas often are divided genetically into two categories: “simple” and “complex.” The simple tumors tend to have specific oncogenic alterations and a limited number of mutations. These include synovial cell sarcoma, which is characterized by a specific translocation [*t*(X;18)(p11;q11)], and liposarcoma, which is characterized by 12q13∼15 amplification. They also include gastrointestinal stromal tumor (GIST), which is characterized by activating mutations of the *KIT* receptor tyrosine kinases. The “complex” tumors, such as undifferentiated pleomorphic and leiomyosarcoma, have numerous genetic mutations but no clear oncogenic driver. The Cancer Genome Atlas Research Network has recently reported on the genomic characteristics of 206 soft tissue sarcomas [7]. Over 3000 soft tissue sarcomas were included among the 100,000 cancers analyzed by a targeted genomic profiling reported by Chalmers et al. [8]. These studies, along with a study reported by Barretina et al. of 207 tumors [9], confirm that the mutational burden in soft tissue sarcoma is not nearly as high as that in traditionally “immunogenic” tumors, such as melanoma. Median somatic rates of approximately 2 per megabase (Mb) of DNA have been observed. In melanoma, this rate is approximately 14 per Mb. Mutational frequencies vary by subtype. Some, including angiosarcoma, leiomyosarcoma, and undifferentiated pleomorphic sarcoma, manifest higher frequencies, in the 2-3 per Mb range. Most, including myxofibrosarcoma, liposarcoma, and synovial sarcoma, manifest very low frequencies, less than 2 per Mb. Nonetheless, patients with high tumor mutational burdens can be identified in nearly every type of soft tissue sarcoma. For example, approximately 13% of angiosarcomas manifest mutation frequencies of more than 20 per Mb [10]. It should be noted that tumor microsatellite instability, which is an indication for treatment with PD-1 inhibitors because of the associated increase in mutations, is not considered to play a major role in soft tissue sarcoma tumorigenesis [9].

The fusion proteins that result from chromosomal translocations represent potential targets. Although there may be tolerance toward epitopes within these proteins, the area of fusion in effect represents a neoantigen. Worley et al. examined the immunogenicity of these areas for sarcoma subtypes characterized by specific translocations, including synovial, clear cell, and desmoplastic round cell [11]. Peptides corresponding to the fusion proteins were designed and assessed for the ability to bind to various HLA class I molecule. These peptides were effectively used *in vitro* to generate antigen-specific T cells with cytotoxic function against tumor cells expressing the fusion protein.

Several soft tissue sarcoma subtypes also naturally have high expression of cancer testis antigens, which are considered targets for immunotherapy, since in adults these proteins are expressed only by germ cells, for example, in testis tissue, and not by somatic tissue cells [11, 12]. NY-ESO-1 is the most notable of the cancer testis antigens described in sarcoma [13, 14]. In synovial sarcoma, NY-ESO-1, as assessed by immunohistochemistry, was expressed in 20 out of 25 cases (80%) [15].

Expression has also been observed in over 90% of myxoid and round-cell liposarcoma. Of note, synovial sarcoma, and myxoid and round-cell liposarcoma are translocation‐driven malignancies with very low mutational burdens.

### 2.2. Tumor Infiltrating Lymphocytes (TILs)

Sarcomas have fewer TILs per gram of tissue and lower ratios of TIL infiltration, when compared to cancers such as melanoma and renal cell carcinoma [16]. In some studies, including studies of patients with GIST, angiosarcoma, leiomyosarcoma, synovial sarcoma, and undifferentiated pleomorphic sarcoma, the presence of TILs has been associated with improved prognosis [17–19]. Other studies of multiple soft tissue sarcoma histologies, including GIST, leiomyosarcoma, and undifferentiated pleomorphic sarcoma, have shown either worse survival or no effect on survival [20–22]. In several cancer types, CD3^+^ and CD8^+^ TILs have been most strongly associated with improved survival [23]. Issels et al. reported on an analysis 341 high-risk localized soft tissue sarcoma patients treated with neoadjuvant chemotherapy and hyperthermia. High TIL counts were associated with enhanced progression-free and disease-free survivals [24].

Sorbye et al. analyzed 249 soft tissue sarcomas, predominantly undifferentiated pleomorphic and liposarcomas, for CD3^**+**^, CD4^**+**^, CD8^**+**^, CD20^**+**^, and CD45^**+**^ lymphocyte infiltration using immunohistochemistry. Only CD20^**+**^ B cells were independently associated with improved disease-free survival [20]. Unexpectedly, low CD3^**+**^ and CD4^**+**^ T-cell infiltrations were associated with better overall survival.

Gene expression profiling has been used to assess intratumoral immune response, and specific signatures that reflect T-cell activation have been shown to have prognostic and/or predictive value [25]. Two recent studies applying molecular techniques have provided more information regarding the immune cell infiltration into soft tissue sarcomas. Pollack et al. performed gene expression and T‐cell receptor V*β* gene sequencing on 81 soft tissue sarcomas [26]. Undifferentiated pleomorphic and leiomyosarcomas had high expression levels of genes related to antigen presentation and T‐cell infiltration. Undifferentiated pleomorphic sarcoma was found to have the highest T‐cell infiltration based on T‐cell receptor sequencing, significantly more than synovial sarcoma, which had the lowest. T‐cell infiltrates in undifferentiated pleomorphic sarcoma also were more oligoclonal compared with synovial sarcoma and liposarcoma. In the Cancer Genome Atlas Research Network study, unsupervised clustering identified variable expression of 203 genes involved in immune response [8]. An immune infiltration score for various immune cells based on their gene expression signatures was developed. CD8 cell score correlated with improved survival in gynecologic leiomyosarcomas. Dendritic cell (DC) scores correlated with improved survival in myxofibrosarcoma/undifferentiated pleomorphic sarcoma, suggesting a role for antigen presentation in the immunologic response to these tumors. Scores related to natural killer (NK) cells correlated with disease-specific survival in leiomyosarcomas and myxofibrosarcoma/undifferentiated pleomorphic sarcoma. The infiltration of NK cells, an antigen-nonspecific immune effector, is rarely noted in human solid tumors, including soft tissue sarcomas [18, 27]. In dedifferentiated liposarcoma, a T-helper 2 signature, which is associated with inhibition of T-cell cytotoxicity, was correlated with shorter disease-specific survival.

### 2.3. Immune Suppressive Mechanisms

The role of specific immune checkpoints, including PD-L1/PD-1, in soft tissue sarcomas is not established. Tumor expression of PD-L1 has been associated with a worse prognosis in most cancers [28]. Kim et al. reported that the expression of PD-L1 by soft tissue sarcomas predicts a poor prognosis [24, 29]. Additionally, the degree of TIL PD-1 positivity showed similar results. D'Angelo et al. noted tumor, lymphocyte, and macrophage PD-L1 expression to be 12%, 30%, and 58%, respectively, with the highest prevalence, 29%, in GIST [30]. In contrast to the work of Kim et al., there was no association between clinical features, overall survival, and PD-L1 expression in tumor or immune infiltrates. Differences have been noted among soft tissue sarcoma subtypes. In the study reported by Pollack et al., undifferentiated pleomorphic sarcoma, which had the highest T‐cell infiltration based on T‐cell receptor sequencing, was found to have higher levels of PD‐L1 and PD‐1 on immunohistochemistry, significantly more than synovial sarcoma, which had the lowest. PD-L1 and PD‐1 expression again was not associated with progression-free of overall survivals. Differences in tumor PD-L1 was also not associated with survivals in the analysis of high-risk localized soft tissue sarcomas performed by Issels et al. [24]. In the Research Network analysis, the highest PD-L1 score, which also was not correlated with survivals, was observed in leiomyosarcoma [8]. Characterization of the immune microenvironment of malignant peripheral nerve sheath tumor resulted in absence of PD1, low PDL1 expression, and minimal CD8 infiltration along with no influence on survival [31].

It has been noted in mouse fibrosarcoma models that the modest antitumor activity of anti-PD-1 therapy was independent of PD-L1 staining [32]. Activity was significantly enhanced when combining anti-PD-1 antibody with antibody against the coinhibitory receptor, LAG-3 [33].

Information regarding the expression in soft tissue sarcoma of LAG-3 and other immune checkpoint ligands and receptors is limited. Differential gene expression was found in the Cancer Genome Atlas Research Network analysis of the coinhibitory ligand B7-H3 and the coinhibitor receptor TIM3, with expression highest in dedifferentiated liposarcoma, undifferentiated pleomorphic sarcoma, and myxofibrosarcoma [8]. Tumors evade macrophage phagocytosis through the expression of antiphagocytic signals within the tumor microenvironment. These include the CD47-SIRP*α* pathway [34]. Antibody therapy targeting the CD47 protein was effective in a metastatic leiomyosarcoma model [35]. TI-621, a recombinant fusion protein that blocks the CD47-SIRP*α* axis, is being tested in clinical trials [34]. The role of tumor suppressor cells is also not established. Tumor-associated macrophages and regulatory T (Treg) cells suppress antitumor immune responses by several mechanisms and have been shown to be negative prognostic factors in several cancers. Tumor-associated macrophages have been associated with negative outcomes in uterine and nonuterine leiomyosarcoma and myxoid liposarcoma [36–38]. The most abundant infiltrating immune cells in GIST include M2 macrophage, an immune-suppressive phenotype [39]. In the Cancer Genome Atlas Research Network analysis, undifferentiated pleomorphic, myxofibrosarcoma, and dedifferentiated liposarcoma had the highest median macrophage scores [40]. Increased recruitment of macrophages in malignant peripheral nerve sheath tumors (MPNSTs) indicate that these tumors may be candidates for response with certain immunotherapy agents [41].

The majority of tumor-infiltrating Treg cells, which express FoxP3, have been detected in GIST rather than non-GIST sarcoma [8]. High FoxP3^**+**^ infiltrates have been reported to correlate with high-risk GIST [42]. Differences in tumor infiltration of FoxP3^+^ cells were not associated with survivals in the analysis of high-risk localized non-GIST soft tissue sarcoma performed by Issels et al.

Several soluble factors have been implicated. The expression of transforming growth factor (TGF) *β*, a cytokine that inhibits antitumor immunity by several mechanisms, has been associated with a poorer survival in soft tissue sarcoma [43]. Higher expression of the immune-suppressive cytokine gene *TGFB1* in undifferentiated pleomorphic, myxofibrosarcoma, and dedifferentiated liposarcoma was noted in the Cancer Genome Atlas Research Network analysis [31]. The proangiogenic cytokine, vascular endothelial growth factor (VEGF), is a significant mediator of immune suppression within the tumor microenvironment, primarily as an inhibitor of DC function. VEGF is frequently overexpressed in soft tissue sarcomas [44].

Indoleamine-2,3-dioxygenase, which catalyzes the oxidative breakdown of the essential amino acid tryptophan, via the kynurenine pathway, is an inhibitor of T-cell proliferation that has been implicated in immune resistance in several cancers, including soft tissue sarcoma [45].

Many of these processes can be operational. Peng et al. recently reported an analysis of the primary tumor and the sole treatment-resistant metastasis of a patient with metastatic uterine leiomyosarcoma who responded to the anti-PD-1 antibody, pembrolizumab. They identified *PTEN* mutations and reduced expression of genes encoding neoantigens as potential mediators of resistance to immune checkpoint therapy [46]. It was noted that both tumors stained diffusely for PD-L2 and showed sparse PD-L1 staining. PD-1^+^ cell infiltration significantly decreased in the resistant tumor. The tumor suppressor *PTEN* in melanoma models leads to immunoresistance by inducing VEGF and other immunosuppressive cytokines [47]. An increase in *VEGFA* gene expression was observed in the treatment-resistant tumor [48].

Finally, biophysical properties of the tumor microenvironment can also promote immunotherapy resistance and immune suppression. The abnormal structure and function of the microvasculature that characterize solid tumors and the increases in tumor interstitial fluid pressure that result in the tumor microenvironment act as a physiological barrier to the delivery of therapeutic agents. Abnormal blood flow can also act as a barrier for immune factors and immune cell migration into the tumor parenchyma. There is evidence that this barrier may contribute to the limited efficacy of immunotherapy [49]. Hypoxia in the tumor microenvironment, in part due to the abnormal vascularity, is considered to play a central role in the suppression of immune effector cells and enhancement of tumor escape from immune surveillance [50]. Soft tissue sarcomas are characterized by high microvascular densities [51], very high tumor interstitial fluid pressures [52], and significant hypoxia [53].

## 3. Intratumoral Immunotherapy

**Figure 1:**
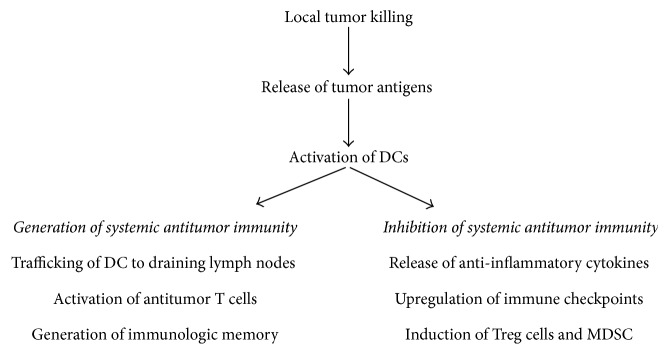
Mechanisms regulating the activity of intratumoral immunotherapy.

The intratumoral administration of immunologically active agents is one approach of directly addressing the limitations presented by the lack of tumor antigenicity and effector lymphocyte infiltration and the multiple immune suppressive mechanisms within the soft tissue sarcoma microenvironment. Intratumoral immunotherapy is not only a method of killing the treated tumor but is also an expedient method of generating systemic antitumor immunity. Immune affector mechanisms as well as immune effector mechanisms can be activated, so that tumor antigens released by tumor dying in situ are processed and presented to expand adaptive, antitumor T cells systemically as well as to generate immunologic memory (Figure 1). Local injections allow much higher concentrations of the immunostimulatory products in the tumor microenvironment than do systemic infusions, which may be important in overwhelming immune suppressor mechanisms. Moreover, local delivery of immunostimulating drugs should prevent their circulation at high concentrations in the blood. Thus, intratumoral immunotherapy should provide improved efficacy and lower toxicity. Intratumoral immunotherapy has been applied to manage accessible lesions and to induce systemic immunity in several cancers. Intratumoral Bacillus Calmette–Guérin (BCG), which has been used for 40 years, is in common use to treat nonmuscle invasive bladder cancer. Recently, an intratumoral therapy with talimogene laherparepvec (T-VEC), an attenuated herpes simplex virus, type 1 (HSV-1) engineered to express human granulocyte-macrophage colony-stimulating factor (GM-CSF), has been approved to treat patients with melanoma.

The use of intratumoral immunotherapy to treat sarcoma is supported not only by the therapeutic observations of Coley and others but also by other clinical observations. Lewis et al. compared 685 sarcoma patients who underwent initial definitive resection to 407 similar patients treated with a definitive re-excision following a previous nontherapeutic excision [54]. Unexpectedly, the 5-year disease-free survival for re-resected patients was significantly higher than the definitive patients, 88% versus 70%. This survival difference could not be explained by a referral bias. When analyzed according to stage, all re-resected patients trended toward an improved outcome in comparison with the definitively treated group. These observations extended beyond the local recurrence-free survival, as there was also an improvement in metastasis-free survival for the re-resected group, suggesting a possible systemic effect. It has been postulated that the local inflammatory response induced with incomplete initial excision may prime an immune response against remaining tumor cells.

Several phenomena may limit the systemic effects of intratumoral immunotherapy (Figure 1). Negative feedback loops designed to alleviate a local inflammatory state may paradoxically cause systemic immunosuppression [55]. The clearance of tumor cells undergoing apoptosis in situ, a process referred to as efferocytosis, is programmed to lead to compartmentalization and anti-inflammatory processing of intracellular self-antigens. Efferocytosis prevents leakage of cytotoxic or antigenic intracellular contents by dying cells and results in the release of anti-inflammatory cytokines, such as TGF-*β*, that suppress the production of proinflammatory mediators locally and systemically, resulting in decreased DC maturation and antigen-specific T cells [56]. Any tissue trauma stimulates inflammatory responses that are highly regulated and result in limiting damage both locally and systemically. Although proportional to the degree of the initial insult, even minor trauma is associated with systemic immune suppression, including decreases in T and NK cell responses. Just as the upregulation of PD-1 has been implicated in inflammation caused by tissue trauma, several soluble mediators, such as TGF-*β* and complement products, and cellular mediators, such as Treg cells and myeloid-derived supressor cells (MDSCs), have been implicated [57, 58].

## 4. Clinical Studies

Whether intratumoral immunotherapy can impact the course of sarcoma has not been established. Mainly pilot and phase I studies involving small numbers of patients have been performed. There are no randomized studies. Nonetheless, antitumor activity has been observed. Multiple approaches are being investigated in preclinical studies in sarcoma models but have yet been advanced to clinical trials. The intratumoral immunotherapy approaches that have been tested clinically or are in the process of being tested clinically are summarized below.

### 4.1. Viral Vectors

Viruses engineered to exploit their inherent antigenicity/immunogenicity as well as to express immunostimulatory molecules are emerging as clinically relevant cancer therapeutics. While direct killing of infected tumor cells is central to their antitumor effect, their ability to enhance immune responses generated to the tumor antigens released through that process is also considered key. Intratumoral therapy with viruses has been shown to induce immune infiltrates not only in the injected tumor but also in distant tumor [59]. Adenoviral vectors, which can accommodate relatively large segments of DNA, have a broad host range and lack pathogenicity, and herpes viruses, which can also accommodate relatively large segments of DNA and are strongly cytolytic in human cancer cells, have been the best studied (Table 1).

Several strategies have been used to enhance the tumor specificity. ONYX-015 is an *E1B-55K* gene-deleted adenovirus. ONYX-015 was originally designed to selectively replicate in and lyse p53-deficient cancer cells [64, 65]. Sarcomas manifest a high frequency of p53 mutations and functional p53 inactivation [66]. However, ONYX-015 was later found to be effective regardless of p53 status, indicating that other mechanisms are responsible for its tumor specificity [67]. ONCOS-102 is an adenovirus with an engineered capsid for enhanced cancer cell transduction and a deletion in the E1A gene, which also promotes proliferation, that binds the tumor suppressor, the retinoblastoma protein (Rb), rendering the viral replication to cells that lack Rb, also commonly observed in soft tissue sarcoma [68, 69]. Because deletion of the RL1 gene and the *γ*_1_34.5 gene, respectively, both which encode virulence factors and the herpes virus vectors HSV1716 and HSV-1 M002, is unable to replicate in nondividing cells, and preferentially infect, replicate in, and lyse rapidly dividing cells such as tumor cells [70, 71].

The incorporation of immune stimulatory cytokines has also been used. GM-CSF, a potent immunostimulatory cytokine that recruits and activates antigen-presenting cells, has been included in several constructs, including ONCOS-102 [68], Ad5/3-D24-GMCSF [72], and JX-594 (pexastimogene devacirepvec), an oncolytic vaccinia virus [73]. TNFerade is a replication-deficient adenovirus that expresses TNF*α*, which not only inhibits tumors directly but also has multiple immune effects, including activation of T cells and DC [74, 75]. The TNF-*α* in TNFerade is expressed downstream of the radiation-inducible Egr-1 promoter gene, which provides spatial and temporal control of the cytotoxicity provided by TNF-*α* when administered intratumorally with radiation [76]. HSV-1 M002 is also engineered to expresses interleukin- (IL-) 12, a major activator of T and NK cells [71].

Several factors can be limiting in viral therapies for cancer. Neutralizing antibodies are highly prevalent and can reduce the efficacy of repeat injections although how antivector immunity influences the clinical or biologic response of intratumoral virotherapy is not known. Extracellular matrix and areas of tissue necrosis, which can be seen in high-grade tumors, may impair the spread of viruses [77]. Hypoxia, a key regulator of the tumor microenvironment, has been shown to decrease infectivity and cytotoxicity of HSV [78]. Macrophages can either support oncolytic virus therapy through proinflammatory stimulation of the antitumor response at the cost of hindering direct oncolysis or through immunosuppressive protection of virus replication at the cost of hindering the antitumor immune response [79].

### 4.2. Immune Cells

Administration of DCs intratumorally has been tested clinically in several cancers [80]. A Phase I study was conducted in 18 patients with high-risk localized soft tissue sarcoma of the intratumoral injection of DCs combined with radiation [81]. An encouraging 11 of 18 (61%) patients were alive with no systemic recurrence over a period of 2 to 8 years. Ten out of 18 (56%) demonstrated evidence of a systemic immune response to either tumor cell lysates or to survivin, a sarcoma-associated antigen. A freeze-stored allogeneic DC preparation, known as INTOVAX, in which DC derived from blood of healthy donors with GM-CSF and IL-4 are 17 activated with toll-like receptor (TLR) 7/8 agonist R848and TLR3 agonist Poly I:C, and human recombinant interferon gamma is under investigation [82].

### 4.3. Cytokines

Cytokines are in common use to treat many malignancies. As noted, viral vectors have been developed to express several cytokines intratumorally, including GM-CSF, TNF, and IL-12. The intratumoral administration of IL-2, a major activator of lymphocyte cytotoxicity, has also been recommended as treatment options for patients with in-transit melanoma metastases. Although tumor regressions were not observed, 6 of fifteen sarcoma patients that were included in a study of the intratumoral administration of direct gene transfer of an IL-2 DNA/DMRIE/DOPE lipid complex had stable disease lasting from 3 to 18 months and continuing [83]. One of the three patients with soft tissue sarcoma-treated IL-2-transfected xenogeneic cells (Vero-IL-2) showed durable reduction of two distant, noninjected metastases [84].

### 4.4. Microbial Products

Intratumoral injections of BCG were tested in patients with sarcoma in several trials conducted in the 1970s [60]. Tumor regressions were also reported with the intratumoral injections of *Corynebacterium parvum*, an approach also tested in the 1970s [61]. The antitumor activity observed was not considered to be sufficient to warrant regulatory approval. Species of *Clostridium* bacteria are notable for their ability to lyse tumor cells growing in hypoxic environments. More recently, spores from an attenuated strain of *Clostridium novyi* (*C. novyi*-NT) have been developed as a therapeutic. A patient with advanced leiomyosarcoma has been reported to respond to an intratumoral injection of *C. novyi*-NT spores [62]. Pathogen-associated molecular patterns (PAMPs), including synthetic constructs that mimic compounds expressed by several types of microbes, are potent immunomodulators. The sequential intratumoral and intramuscular injections of the synthetic PAMP, polyinosinic-polycytidylic acid-polylysine-carboxymethylcellulose (Poly-ICLC), has been reported to be active in sarcoma [63].

## 5. Future Prospects

The rationale as well as the feasibility and safety for the intratumoral injection of immunotherapeutics into sarcoma tumors has been established in several studies. Several clinical trials are in progress (Table 2).

Further clinical investigation is needed to better define how the expression and presentation of intratumoral antigens are regulated in soft tissue sarcomas. There remain many unanswered questions about which immune cells may dictate prognosis, and no available data to date correlating immune infiltration with response to modern immunotherapy in sarcoma. The immune checkpoints operational in soft tissue sarcoma progression are not established. A better assessment of particular T-cell phenotypes, activation status, and the presence of other suppressive immune cells and factors is needed to optimize intratumoral immunotherapy. Although generating systemic antitumor immunity is a major goal of intratumoral immunotherapy, very few studies have examined systemic immune responses. Neoadjuvant approaches in patients with high-risk localized soft tissue sarcomas considered candidates for surgery would be an ideal system to examine these.

There is little to suggest that intratumoral immunotherapy that focuses only on one aspect of immune effector activation will be highly effective either locally or systemically. Single agent therapy is usually not completely effective, even in preclinical models. Studies of treatments in which mechanistically distinct immunotherapeutics are combined need to continue. Systemic chemotherapy has been shown to increase the antitumor activity of intratumoral immunotherapy [85, 86]. Radiation has also been effectively applied to enhance intratumoral immunotherapy [87]. There are many questions that need to be addressed regarding the immune effects of chemotherapy and radiation in patients with soft tissue sarcoma. Other combination approaches the merit study. Efferocytosis may be modifiable [56]. Approaches to disrupt the sarcoma stroma by targeting, for example, matrix metalloproteinases, angiogenic factors, and hyaluronic acid, and to improve tumor oxygenation are under investigation [88]. These approaches may have application to immunotherapeutics.

Since conventional computed tomography (CT) or magnetic resonance imaging (MRI) alone may not be adequate to determine immunotherapy response, the utility of more functional imaging, such as positron emission tomography, diffusion-weighted imaging, dynamic contrast-enhanced magnetic resonance imaging, and perfusion computed tomography, should be investigated. Furthermore, fine-needle injection expertise is not uniformly available, even among cancer centers. Development of specific devices, including needles suited for the administration of immunologic agents, is another issue to be addressed. Newer systems, such as three-dimensional ultrasound-CT dual imaging, should be tested to plan and to monitor immune drug delivery.

Finally, novel animal models are needed. Most studies have been performed using chemically induced tumors in mice. The immune system of rodents, however, has well recognized differences from that of humans. It should be noted that most studies applied intratumoral treatments to subcutaneous and not orthotopic tumors. It is not clear that the microenvironment of even orthotopic rodent sarcoma mimics the human situation. Immunotherapy studies require mouse models with an intact immune system. Immunocompromised xenogeneic mouse models with transplantable human sarcoma cells and cell lines do not allow for assessment of antitumor immune responses but can provide information regarding, for example, the pharmacokinetics of the intratumoral injection. Humanized mouse models have been developed to allow the study of xenografts in the context of a human immune system [89]. Genetically engineered mouse sarcoma models have been developed. The processes involved in developing these models, however, also significantly alter immunogenicity. The caveat here is that unless the tumor and marrow come from the same patient, there will be HLA mismatch and thus will not recapitulate immunologic fidelity. Several genetically engineered models have recently been created for GIST, liposarcomas, and some translocation-related sarcomas. For a number of sarcoma subtypes, including undifferentiated sarcomas and angiosarcomas, very few or no models are available [90].

## 6. Conclusions

Therapy of soft tissue sarcomas represents an area of significant unmet need in oncology. Systemic immunotherapies have failed to demonstrate significant clinical activity due to a tumor microenvironment characterized by low tumor antigenicity, limited infiltration of effector cells, and several processes that suppress immune cell function. Intratumoral immunotherapy can enhance tumor antigenicity, promote TILs, and generate a systemic antitumor immune response. Because tumor is often easily accessible and immune mechanisms are implicated in regulating its progression, the intratumoral application of immune modulators has been an attractive treatment for sarcoma. Although intratumoral immunotherapy has not yet been established as a standard option in the treatment in sarcoma, its feasibility, safety, and biologic activity have been proven in clinical trials. The rationale design of immunotherapy strategies will require improved understanding of the regulation of immune responses in soft tissue microenvironment. Given Coley's early foray, it has been disappointing that research into the immune biology of soft tissue sarcoma has been slower than in other cancers. Progress has recently been made. Novel, more effective immunotherapeutic strategies should be forthcoming.

## Figures and Tables

**Table 1:** Clinical trials of intratumoral viral vector therapy in soft tissue sarcoma.

Agent	*N*	Results	Ref.
TNFerade	14	Of 13 evaluable patients, 11 (85%) objective or pathological tumor responses (2 CR and 9 PR), 1 SD	[60]
ONYX-015 (+mitomycin-C, doxorubicin, cisplatin)	6	ONYX-015 viral DNA detected in 2 patient biopsies and 5 patient plasma after treatment	[61]
Ad5/3-D24-GMCSF	15	2 minor responses, 6 SD, and 4 PD in 12 evaluable patients; median survival time after treatment was 170 days. One patient was alive at 1459 days	[62]
HSV1716	9	Four of 5 patients evaluated at day +14 had stable disease by cross-sectional imaging. Three of 7 patients evaluated at day +28 had stable disease, and one of these patients had a decrease in PET standardized uptake values	[63]

**Table 2:** Currently recruiting clinical trials of intratumoral immunotherapy in soft tissue sarcoma.

Treatment	Phase	ClinicalTrials.gov identifier
HSV-1 M002 (IT or IV)	I	NCT00931931
HSV-1 M002 + concurrent radiation	I/II	NCT02453191
HSV-1 M002 + pembrolizumab	II	NCT03069378
TVEC + preoperative radiation	I/II	NCT02453191
JX-594 + cyclophosphamide	I/II	NCT02630368
TTI-621	I	NCT02890368
INTUVAX	I	NCT02686944
Poly-ICLC	II	NCT01984892
*Clostridium novyi*-NT spores	I	NCT01924689

## References

[1] Jacobs A. J., Michels R., Stein J., Levin A. S. (2015). Improvement in overall survival from extremity soft tissue sarcoma over twenty years. *Sarcoma*.

[2] Coley W. B. (1891). Contribution to the knowledge of sarcoma. *Annals of Surgery*.

[3] Maki R. G., Jungbluth A. A., Gnjatic S. (2013). A pilot study of anti-CTLA4 antibody ipilimumab in patients with synovial sarcoma. *Sarcoma*.

[4] D’Angelo S. P., Shoushtari A. N., Keohan M. L. (2017). Combined KIT and CTLA-4 blockade in patients with refractory GIST and other advanced sarcomas: a phase Ib study of dasatinib plus ipilimumab. *Clinical Cancer Research*.

[5] Tawbi H. A., Burgess M., Bolejack V. (2017). Pembrolizumab in advanced soft-tissue sarcoma and bone sarcoma (SARC028): a multicentre, two-cohort, single-arm, open-label, phase 2 trial. *The Lancet Oncology*.

[6] Toulmonde M., Penel N., Adam J. (2018). Use of PD-1 targeting, macrophage infiltration, and IDO pathway activation in sarcomas: a phase 2 clinical trial. *JAMA Oncology*.

[7] Cancer Genome Atlas Research Network (2017). Electronic address: cancer genome atlas research network. Comprehensive and integrated genomic characterization of adult soft tissue sarcomas. *Cell*.

[8] Chalmers Z. R., Connelly C. F., Fabrizio D (2017). Analysis of 100,000 human cancer genomes reveals the landscape of tumor mutational burden. *Genome Medicine*.

[9] Barretina J., Taylor B. S., Banerji S. (2010). Subtype-specific genomic alterations define new targets for soft-tissue sarcoma therapy. *Nature Genetics*.

[10] Campanella N. C., Penna V., Ribeiro G., Abrahão-Machado L. F., Scapulatempo-Neto C, Reis R. M. (2015). Absence of microsatellite instability in soft tissue sarcomas. *Pathobiology*.

[11] Worley B. S., van den Broeke L. T., Goletz T. J. (2001). Antigenicity of fusion proteins from sarcoma-associated chromosomal translocations. *Cancer Research*.

[12] Robbins P. F., Kassim S. H., Tran T. L. (2015). A pilot trial using lymphocytes genetically engineered with an NY-ESO-1-reactive T-cell receptor: long-term follow-up and correlates with response. *Clinical Cancer Research*.

[13] Somaiah N., Block M. S., Kim J. W. (2015). Phase I, first-in-human trial of LV305 in patients with advanced or metastatic cancer expressing NY-ESO-1. *Journal of Clinical Oncology*.

[14] Jungbluth A. A., Antonescu C. R., Busam K. J. (2001). Monophasic and biphasic synovial sarcomas abundantly express cancer/testis antigen NY-ESO-1 but not MAGE-A1 or CT7. *International Journal of Cancer*.

[15] Pollack S. M., Jungbluth A. A., Hoch B. L. (2012). NY-ESO-1 is a ubiquitous immunotherapeutic target antigen for patients with myxoid/round cell liposarcoma. *Cancer*.

[16] Hemminger J. A., Iwenofu O. H. (2013). NY-ESO-1 is a sensitive and specific immunohistochemical marker for myxoid and round cell liposarcomas among related mesenchymal myxoid neoplasms. *Modern Pathology*.

[17] Balch C. M., Riley L. B., Bae Y. J. (1990). Patterns of human tumor-infiltrating lymphocytes in 120 human cancers. *Archives of Surgery*.

[18] Rusakiewicz S., Semeraro M., Sarabi M. (2013). Immune infiltrates are prognostic factors in localized gastrointestinal stromal tumors. *Cancer Research*.

[19] Fujii H., Arakawa A., Utsumi D. (2014). CD8+ tumor-infiltrating lymphocytes at primary sites as a possible prognostic factor of cutaneous angiosarcoma. *International Journal of Cancer*.

[20] Sorbye S. W., Kilvaer T., Valkov A. (2011). Prognostic Impact of Lymphocytes in soft tissue sarcomas. *PLoS One*.

[21] D’Angelo S. P., Shoushtari A. N., Agarm N. P. (2014). Prevalence of tumor-infiltrating lymphocytes and PD-L1 expression in the soft tissue sarcoma microenvironment. *Human Pathology*.

[22] Kim C., Kim E. K., Jung H. (2016). Prognostic implications of PD-L1 expression in patients with soft tissue sarcoma. *BMC Cancer*.

[23] Gooden M. J., de Bock G. H., Leffers N., Daemen T., Nijman H. W. (2011). The prognostic influence of tumour-infiltrating lymphocytes in cancer: a systematic review with meta-analysis. *British Journal of Cancer*.

[24] Issels R., Büclein V., Kampmann E. (2016). Dissecting the role of tumor-infiltrating lymphocytes (TIL) in patients with high-risk soft-tissue sarcoma (STS) receiving neo-adjuvant chemotherapy (NAC) with regional hyperthermia (RHT). *Annals of Oncology*.

[25] Iglesia M. D., Parker J. S., Hoadley K. A., Serody J. S., Perou C. M., Vincent B. G. (2016). Genomic analysis of immune cell infiltrates across 11 tumor types. *Journal of the National Cancer Institute*.

[26] Pollack S. M., He Q., Yearley J. H. (2017). T-cell infiltration and clonality correlate with programmed cell death protein 1 and programmed death-ligand 1 expression in patients with soft tissue sarcomas. *Cancer*.

[27] Desbois M., Rusakiewicz S., Locher C., Zitvogel L., Chaput N. (2012). Natural killer cells in non-hematopoietic malignancies. *Frontiers in Immunology*.

[28] Wu P., Wu D., Li L., Chai Y., Huang J. (2015). PD-L1 and survival in solid tumors: a meta-analysis. *PLoS One*.

[29] Kim J. R., Moon Y. J., Kwon K. S. (2013). Tumor infiltrating PD1-positive lymphocytes and the expression of PD-L1 predict poor prognosis of soft tissue sarcomas. *PLoS One*.

[30] D’Angelo S. P., Shoushtari A. N., Agarm N. P. (2014). Prevalence of tumor-infiltrating lymphocytes and PD-L1 expression in the soft tissue sarcoma microenvironment. *Human Pathology*.

[31] Shurell E., Singh A. S., Crompton J. G. (2016). Characterizing the immune microenvironment of malignant peripheral nerve sheath tumor by PD-L1 expression and presence of CD8+ tumor infiltrating lymphocytes. *Oncotarget*.

[32] Wei S., Shreiner A. B., Takeshita N., Chen L., Zou W., Chang A. E. (2008). Tumor-induced immune suppression of in vivo effector T-cell priming is mediated by the B7-H1/PD-1 axis and transforming growth factor beta. *Cancer Research*.

[33] Woo S. R., Turnis M. E., Goldberg M. V. (2012). Immune inhibitory molecules LAG-3 and PD-1 synergistically regulate T-cell function to promote tumoral immune escape. *Cancer Research*.

[34] Petrova P. S., Viller N. N., Wong M. (2017). TTI-621 (SIRP*α*Fc): a CD47-blocking innate immune checkpoint inhibitor with broad antitumor activity and minimal erythrocyte binding. *Clinical Cancer Research*.

[35] Soto-Pantoja D. R., Terabe M., Ghosh A. (2014). CD47 in the tumor microenvironment limits cooperation between antitumor T- cell immunity and radiotherapy. *Cancer Research*.

[36] Edris B., Weiskopf K., Volkmer A. K. (2012). Antibody therapy targeting the CD47 protein is effective in a model of aggressive metastatic leiomyosarcoma. *Proceedings of the National Academy of Sciences*.

[37] Espinosa I., Beck A. H., Lee C. H. (2009). Coordinate expression of colony-stimulating factor-1 and colony-stimulating factor-1-related proteins is associated with poor prognosis in gynecological and nongynecological leiomyosarcoma. *American Journal of Pathology*.

[38] Lee C. H., Espinosa I., Vrijaldenhoven S. (2008). Prognostic significance of macrophage infiltration in leiomyosarcomas. *Clinical Cancer Research*.

[39] van Dongen M., Savage N. D., Jordanova E. S. (2010). Anti-inflammatory M2 type macrophages characterize metastasized and tyrosine kinase inhibitor-treated gastrointestinal stromal tumors. *International Journal of Cancer*.

[40] Nabeshima A., Matsumoto Y., Fukushi J. (2015). Tumour-associated macrophages correlate with poor prognosis in myxoid liposarcoma and promote cell motility and invasion via the HB-EGF-EGFR-PI3K/Akt pathways. *British Journal of Cancer*.

[41] Jour G., Andeen N. K., Al-Rohil R. (2018). Novel enriched pathways in superficial malignant peripheral nerve sheath tumours and spindle/desmoplastic melanomas. *Journal of Pathology*.

[42] Tan T., Trent J. C., Wilky B. A., Kerr D. A., Rosenberg A. E. (2017). Current status of immunotherapy for gastrointestinal stromal tumor. *Cancer Gene Therapy*.

[43] Valkov A., Sorbye S. W., Kilvaer T. K. (2011). The prognostic impact of TGF-β1, fascin, NF-κB and PKC-ζ expression in soft tissue sarcomas. *PLoS One*.

[44] DuBois S., Demetri G. (2007). Markers of angiogenesis and clinical features in patients with sarcoma. *Cancer*.

[45] Balachandran V. P., Cavnar M. J., Zeng S. (2011). Imatinib potentiates antitumor T cell responses in gastrointestinal stromal tumor through the inhibition of Ido. *Nature Medicine*.

[46] George S., Miao D., Demetri G. D. (2017). Loss of PTEN Is Associated with Resistance to Anti-PD-1 Checkpoint Blockade Therapy in Metastatic Uterine Leiomyosarcoma. *Immunity*.

[47] Peng W., Chen J. Q., Liu C. (2016). Loss of PTEN promotes resistance to T cell-mediated immunotherapy. *Cancer Discovery*.

[48] Toso A., Revandkar A., Di Mitri D. (2014). Enhancing chemotherapy efficacy in Pten-deficient prostate tumors by activating the senescence-associated antitumor immunity. *Cell Reports*.

[49] Manzur M., Hamzah J., Ganss R. (2008). Modulation of the “blood-tumor” barrier improves immunotherapy. *Cell Cycle*.

[50] Lee C. T., Mace T., Repasky E. A. (2010). Hypoxia-driven immunosuppression: a new reason to use thermal therapy in the treatment of cancer?. *International Journal of Hyperthermia*.

[51] West C. C., Brown N. J., Mangham D. C., Grimer R. J., Reed M. W. (2005). Microvessel density does not predict outcome in high grade soft tissue sarcoma. *European Journal of Surgical Oncology*.

[52] Stohrer M., Boucher Y., Stangassinger M., Jain R. K. (2000). Oncotic pressure in solid tumors is elevated. *Cancer Research*.

[53] Nordsmark M., Alsner J., Keller J. (2001). Hypoxia in human soft tissue sarcomas: adverse impact on survival and no association with p53 mutations. *British Journal of Cancer*.

[54] Lewis J. J., Leung D., Espat J., Woodruff J. M., Brennan M. F. (2000). Effect of reresection in extremity soft tissue sarcoma. *Annals of Surgery*.

[55] Balkwill F., Mantovani A. (2001). Inflammation and cancer: back to Virchow?. *The Lancet*.

[56] Vandivier R. W., Henson P. M., Douglas I. S. (2006). Burying the dead: the impact of failed apoptotic cell removal (efferocytosis) on chronic inflammatory lung disease. *Chest*.

[57] Triozzi P. L., Tuthill R. J., Borden E. (2011). Re-inventing intratumoral immunotherapy for melanoma. *Immunotherapy*.

[58] Arai Y., Saito H., Ikeguchi M. (2012). Upregulation of TIM-3 and PD-1 on CD4+ and CD8+ T cells associated with dysfunction of cell-mediated immunity after colorectal cancer operation. *Yonago Acta Medica*.

[59] Zamarin D., Holmgaard R. B., Subudhi S. K. (2014). Localized oncolytic virotherapy overcomes systemic tumor resistance to immune checkpoint blockade immunotherapy. *Science Translational Medicine*.

[60] Morton D. L. (1972). Immunotherapy of human melanomas and sarcomas. *National Cancer Institute Monograph*.

[61] Cheng V. S., Suit H. D., Wang C. C., Raker J., Weymuller E., Kaufman S. (1978). A preliminary study of intralesional, intralymph node, intravenous and intraperitoneal Corynebacterium parvum treatments in patients with advanced cancer. *Cancer*.

[62] Roberts N. J., Zhang L., Janku F. (2014). Intratumoral injection of Clostridium novyi-NT spores induces antitumor responses. *Science Translational Medicine*.

[63] Salazar A. M., Erlich R. B., Mark A., Bhardwaj N., Herberman R. B. (2014). Therapeutic in situ autovaccination against solid cancers with intratumoral poly-ICLC: case report, hypothesis, and clinical trial. *Cancer Immunology Research*.

[64] Wodarz D. (2001). Viruses as antitumor weapons: defining conditions for tumor remission. *Cancer Research*.

[65] Kirn D. (2001). Clinical research results with dl1520 (ONYX-015), a replication-selective adenovirus for the treatment of cancer: what have we learned?. *Gene Therapy*.

[66] Seki A., Kodama J., Miyagi Y., Kamimura S., Yoshinouchi M., Kudo T. (1997). Amplification of the mdm-2 gene and p53 abnormalities in sarcomas. *International Journal of Cancer*.

[67] Goodrum F. D., Ornelles D. A. (1998). p53 status does not determine outcome of E1B 55-kilodalton mutant adenovirus lytic infection. *Journal of Virology*.

[68] Ranki T., Pesonen S., Hemminki A. (2016). Phase I study with ONCOS-102 for the treatment of solid tumors - an evaluation of clinical response and exploratory analyses of immune markers. *Journal for ImmunoTherapy of Cancer*.

[69] Karpeh M. S., Brennan M. F., Cance W. G. (1995). Altered patterns of retinoblastoma gene product expression in adult soft-tissue sarcomas. *British Journal of Cancer*.

[70] Streby K. A., Geller J. I., Currier M. A. (2017). Intratumoral injection of HSV1716, an oncolytic herpes virus, is safe and shows evidence of immune response and viral replication in young cancer patients. *Clinical Cancer Research*.

[71] Markert J. M., Cody J. J., Parker J. N. (2012). Preclinical evaluation of a genetically engineered herpes simplex virus expressing interleukin-12. *Journal of Virology*.

[72] Bramante S., Koski A., Kipar A. (2014). Serotype chimeric oncolytic adenovirus coding for GM-CSF for treatment of sarcoma in rodents and humans. *International Journal of Cancer*.

[73] Cripe T. P., Ngo M. C., Geller J. I. (2015). Phase 1 study of intratumoral Pexa-Vec (JX-594), an oncolytic and immunotherapeutic vaccinia virus, in pediatric cancer patients. *Molecular Therapy*.

[74] Brunner C., Seiderer J., Schlamp A. (2000). Enhanced dendritic cell maturation by TNF-alpha or cytidine-phosphate-guanosine DNA drives T cell activation in vitro and therapeutic anti-tumor immune responses in vivo. *Journal of Immunology*.

[75] MacGill R. S., Davis T. A., Macko J., Mauceri H. J., Weichselbaum R. R., King C. R. (2007). Local gene delivery of tumor necrosis factor alpha can impact primary tumor growth and metastases through a host-mediated response. *Clinical and Experimental Metastasis*.

[76] Hallahan D. E., Mauceri H. J., Seung L. P. (1995). Spatial and temporal control of gene therapy using ionizing radiation. *Nature Medicine*.

[77] Haseley A., Boone S., Wojton J. (2012). Extracellular matrix protein CCN1 limits oncolytic efficacy in glioma. *Cancer Research*.

[78] Friedman G. K., Nan L., Haas M. C. (2015). *γ*134.5-deleted HSV-1-expressing human cytomegalovirus IRS1 gene kills human glioblastoma cells as efficiently as wild-type HSV-1 in normoxia or hypoxia. *Gene Therapy*.

[79] Denton N. L., Chen C. Y., Scott T. R., Cripe T. P. (2016). Tumor-associated macrophages in oncolytic virotherapy: friend or foe?. *Biomedicines*.

[80] Triozzi P. L., Khurram R., Aldrich W. A., Walker M. J., Kim J. A., Jaynes S. (2000). Intratumoral injection of dendritic cells derived in vitro in patients with metastatic cancer. *Cancer*.

[81] Raj S., Bui M. M., Springett G. (2015). Long-term clinical responses of neoadjuvant dendritic cell infusions and radiation in soft tissue sarcoma. *Sarcoma*.

[82] Laurell A., Lönnemark M., Brekkan E. (2017). Intratumorally injected pro-inflammatory allogeneic dendritic cells as immune enhancers: a first-in-human study in unfavourable risk patients with metastatic renal cell carcinoma. *Journal for ImmunoTherapy of Cancer*.

[83] Galanis E., Hersh E. M., Stopeck A. T. (1999). Immunotherapy of advanced malignancy by direct gene transfer of an interleukin-2 DNA/DMRIE/DOPE lipid complex: phase I/II experience. *Journal of Clinical Oncology*.

[84] Rochlitz C., Jantscheff P., Bongartz G. (1999). Gene therapy study of cytokine-transfected xenogeneic cells (Vero-interleukin-2) in patients with metastatic solid tumors. *Cancer Gene Therapy*.

[85] Zhong H., Han B., Tourkova I. L. (2007). Low-dose paclitaxel prior to intratumoral dendritic cell vaccine modulates intratumoral cytokine network and lung cancer growth. *Clinical Cancer Research*.

[86] Fridlender Z. G., Sun J., Singhal S. (2010). Chemotherapy delivered after viral immunogene therapy augments antitumor efficacy via multiple immune-mediated mechanisms. *Molecular Therapy*.

[87] Brody J. D., Ai W. Z., Czerwinski D. K. (2010). In situ vaccination with a TLR9 agonist induces systemic lymphoma regression: a phase I/II study. *Journal of Clinical Oncology*.

[88] Gkretsi V., Stylianou A., Papageorgis P., Polydorou C., Stylianopoulos T. (2015). Remodeling components of the tumor microenvironment to enhance cancer therapy. *Frontiers in Oncology*.

[89] Seitz G., Pfeiffer M., Fuchs J. (2010). Establishment of a rhabdomyosarcoma xenograft model in human-adapted mice. *Oncology Reports*.

[90] Hamacher R., Bauer S. (2017). Preclinical models for translational sarcoma research. *Current Opinion in Oncology*.

